# Computed tomography: Diagnostic detection of complete pericardial agenesis: A case report

**DOI:** 10.1016/j.radcr.2024.02.102

**Published:** 2024-03-23

**Authors:** Cesare Oliveti, Nicoletta Signati, Claudia Italia Maria De Santis, Roberta Mancini, Danilo Flauti, Giuseppe Lucio Cascini

**Affiliations:** a Division of Radiology, Department of Experimental and Clinical Medicine, Azienda Ospedaliero-Universitaria Renato Dulbecco, Catanzaro, Italy; b Department of Diagnostic Imaging, Nuclear Medicine Unit, Azienda Ospedaliero-Universitaria Renato Dulbecco, Catanzaro, Italy

**Keywords:** Computed tomography, Magnetic resonance imaging, Complete pericardial agenesis, Pericardium

## Abstract

Congenital complete absence of the pericardium is a rare condition, often difficult to diagnose due to its incidental discovery or nonspecific clinical manifestations. Instrumental investigations commonly used as initial approaches, such as chest radiography and electrocardiogram, are often insufficient. Echocardiography is an imaging technique that is used for the initial evaluation of pericardial diseases. However, echocardiography does not offer a physiological anatomical delineation of the pericardium and can be affected by operator dependency, acoustic and nontraditional imaging windows. Therefore, accurate imaging techniques such as computed tomography (CT) or magnetic resonance imaging (MRI) are required for correct diagnosis. We present a case of a symptomatic patient with complete pericardial agenesis diagnosed on angio-CT. This case can contribute to highlighting the importance of CT as a comprehensive imaging method in diagnosis, despite MRI being the gold standard in pericardial disease assessment.

## Introduction

The absence of the pericardium was first suggested by the Italian anatomist and surgeon M. Realdus Columbus in 1559. The incidence of pericardium agenesis is approximately 0.004%, as defined by the studies conducted by Ellis et al. in 1959. Pericardial defects are categorized into 6 categories: right (partial or complete), left (partial or complete), total absence, and diaphragmatic defects. Total absence of both right and left pericardium is the rarest of all defects, observed in only 9% of cases reported in the literature, and may present either in isolated form or in association with other congenital anomalies [1].

In this case report, we present a case of *patient with a rare congenital pathology, complete pericardial agenesis, incidental diagnosed by angio-CT.*

## Case presentation

A 62-year-old male was admitted to our medical facility in June 2023 due to intermittent chest pain, dyspnea, and asthenia. His medical history was positive for dyslipidemia and hypertension, with an occurrence of acute coronary syndrome (ACS) in 2010, managed through primary coronary angioplasty with stenting (PTCA) of the anterior interventricular branch. The patient underwent comprehensive hematological examinations, electrocardiogram (ECG), echocardiogram, and chest radiography.

**Fig. 1:**
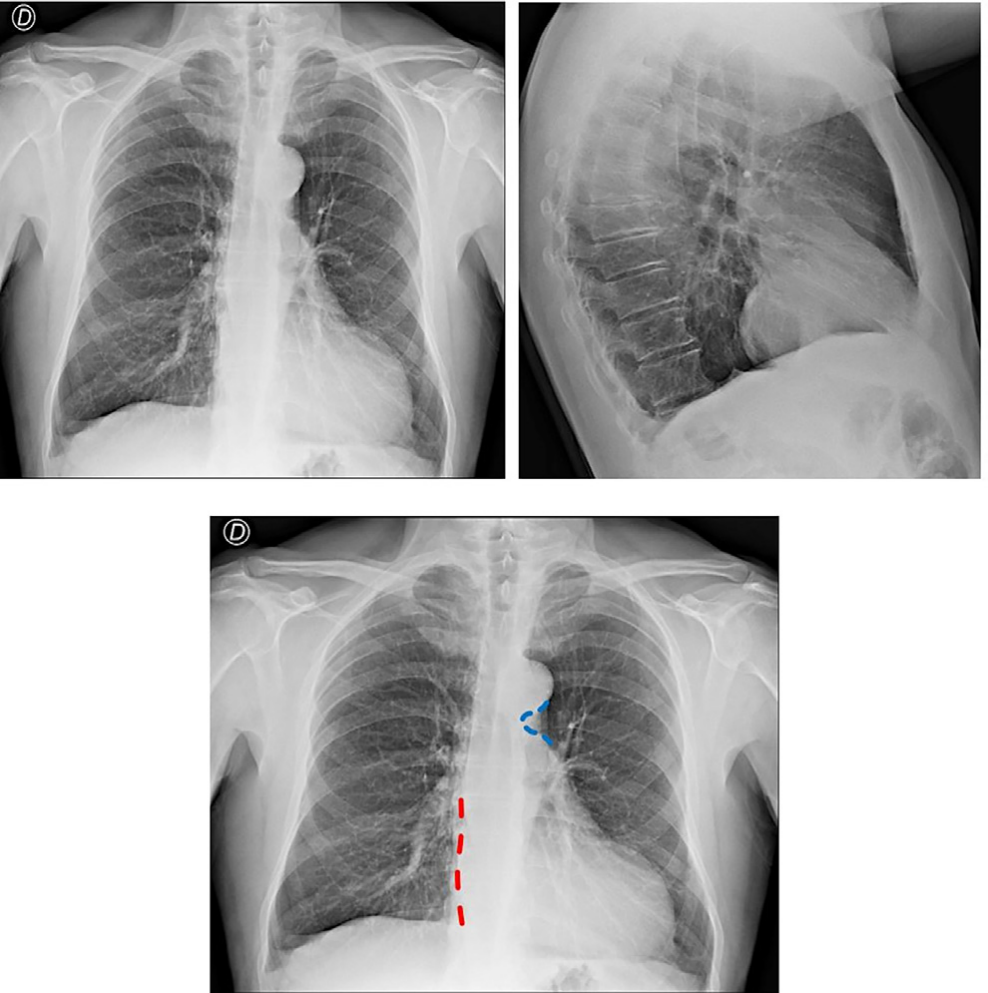
(A) Chest X-ray postero-anterior (PA) and lateral-lateral (LL) projections reported as negative; (B) Chest X-ray *featuring a levopositioned heart with a loss of definition of the rigth second arc border (red dashed line) and a small aortic knuckle with prominent notch (blue dashed line)*.

The outcomes of hematological assessments were insignificant. The ECG depicted a sinus rhythm, with ventricular conduction approaching the upper limits of normal (PR 200 msec), right-axis QRS deviation, right bundle branch block, and nonspecific ventricular repolarization changes. The technically challenging echocardiogram revealed ascending aorta ectasia, normal dimensions of the aortic root and left ventricle, increased parietal thickness, and diminished indices of left ventricular systolic function (EF 40%). These findings were indicative of ischemic heart disease with compromised systolic function and aortic ectasia. Chest radiography yielded unremarkable results (Fig. 1A).

**Fig. 2:**
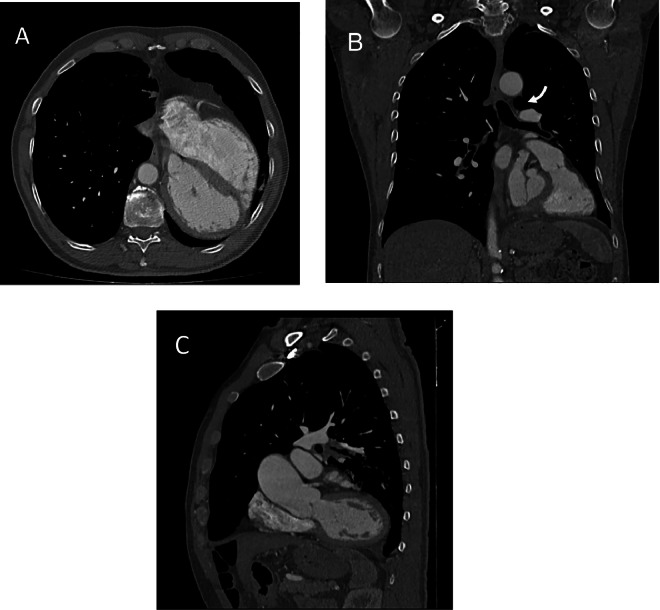
Cardiac computed tomography imaging demonstrating complete pericardial agenesis (A-C): *(A) Transaxial section; noticeable levorotation and levoposition of the heart (B) Coronal section; a conspicuous notch between the aorta and the pulmonary artery (arrow) (C) Sagittal section; posterior position of the apex*.

It was deemed necessary to further elucidate aortic pathology through chest angio-CT, before and after organ iodinated contrast medium administration. Imaging was achieved employing a 64-detector multidetector CT, incorporating a gated CTA protocol aimed at optimizing material contrast and concurrently minimizing aortic wall motion, radiation exposure, and intravenous contrast volume. The protocol encompassed an initial pre-contrast scan, succeeded by a prompt injection of contrast medium (4 mL/sec) with a concentration of approximately 400 mg/mL, aimed at maximizing aortic enhancement during the arterial phase. Thin-section reconstructions (1-2 mm) were employed. The CT demonstrated levorotation and levoposition of the heart with displacement of the left ventricular apex, coupled with interposition of lung tissue between the main pulmonary artery and the ascending aorta, culminating in the diagnosis of complete pericardial agenesis (Fig. 2).

## Discussion

The pericardium originates from 1 of the 3 intraembryonic coelomic cavities during the fourth week of embryogenesis. Failure of fusion of the pericardial cavities can result in congenital agenesis of the pericardium [2].

Complete agenesis of the pericardium is often silent, and if symptoms are present, they are nonspecific and can mimic many other conditions: acute coronary syndromes, pericarditis, myocarditis, cardiac aneurysms, lung or heart tumors, mitral valve diseases, atrial septal defects, pulmonary stenosis, idiopathic pulmonary artery dilation, and hilar lymphadenopathy.

In patients with congenital absence of the pericardium, physical examination is unremarkable. Electrocardiogram may identify cardiac axis deviation, but it is not sufficient for diagnosis.

Echocardiography is the imaging technique of choice for the initial evaluation of pericardial diseases because it is easily accessible and does not use ionizing radiation. The use of nontraditional cardiac windows in this methodology may lead to suspicion due to the detection of leftward positioning and hypermobility of the heart (cardiopexy). This imaging technique cannot be used to analyze the entire pericardium due to the intrinsic limitations of the acoustic window.

Cardiac computed tomography (CCT) and cardiac magnetic resonance imaging (CMR) are now the preferred diagnostic modalities for resolving these limitations. CMR is accepted as the gold standard because it provides a complete representation of the pericardium and adequate functional assessment without the use ionizing radiation [3], [4], [5]***.***

**Fig. 3:**
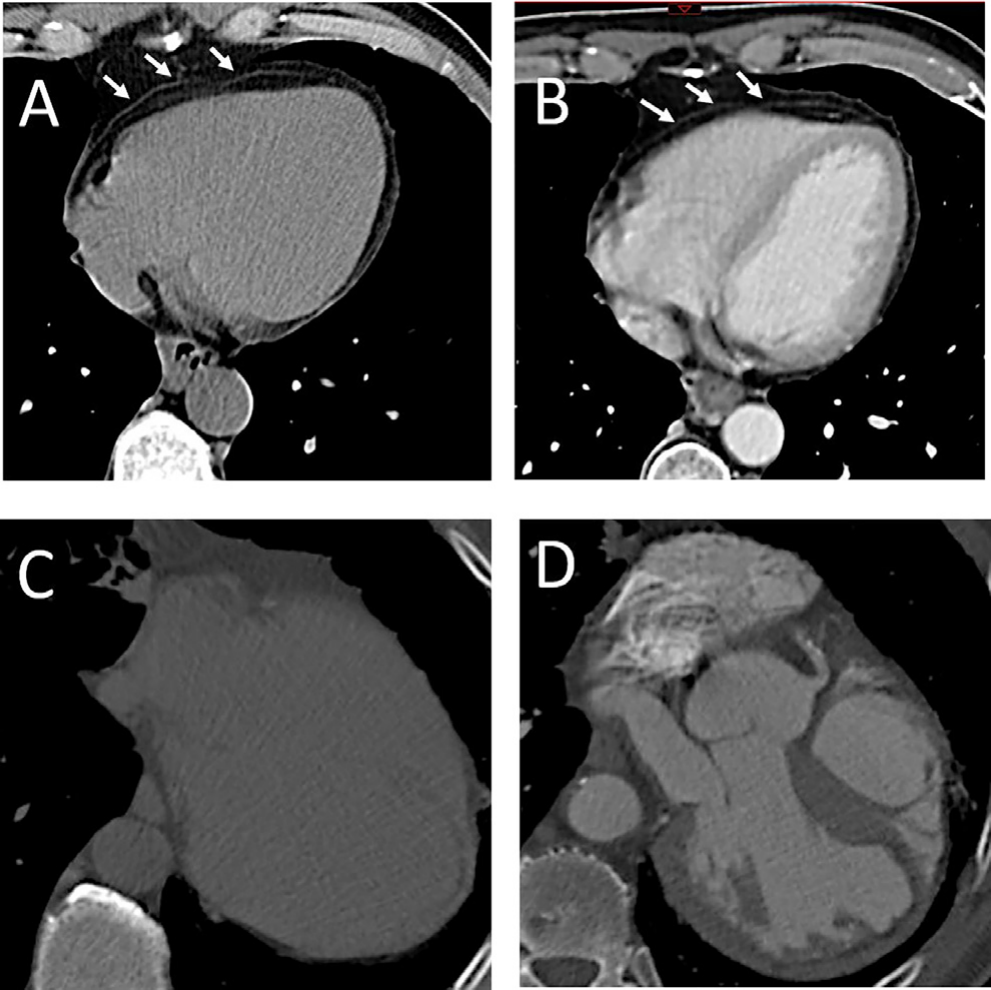
Cardiac computed tomography imaging normal pericardium and congenital absence (A, C) *without contrast agent,* (B, D) *with contrast agent: (A, B) Transaxial section show the pericardium like linear band with a thickness of less than 2 mm (arrow); (C, D) Transaxial section the linear band is not apparent due to the absence of the pericardium.*

But CT like CMR offers an anatomical delineation of the pericardium, allowing for accurate measurement of its thickness (Fig. 3). The visibility of the pericardium in the CT examination is enhanced against the low attenuation of surrounding adipose tissue and is subject to limitations similar to CMR [6]. CT facilitates pericardial visualization in images without contrast and contrast enhancement, reducing motion artifacts through electrocardiographic gating, and using multiplanar reconstruction improves visualization. [4,5]***.***

The CT signs that point to a congenital pericardial absence include: interposition of lung tissue between the aorta and the main pulmonary artery, protrusion of the left atrial appendage through the defect and the heart usually rotates to the left [7].

Additionally, CT like CMR can highlight associated congenital anomalies such as atrial septal defects, patent ductus arteriosus, mitral valve stenosis, or tetralogy of Fallot. CT's rapid execution and superior spatial resolution make it ideal for less compliance patients and overcome the temporal restrictions of CMR. A prospective protocol can be used to obtain optimal images while minimizing exposure to ionizing radiation.

In summary, computed tomography can make morphological and functional evaluations similar to CMR, ensuring diagnostic accuracy [4,5]***.*** This also explains why, according to a multisociety consensus statement on a scale from 1 to 9 (1 being the least appropriate and 9 being the most appropriate), both CT and MRI receive a score of 8 for the assessment of pericardial diseases [8].

However, congenital pericardial absence can be suspected with chest radiography, as characteristic findings may be recognized: leftward position cardiac of silhouette in posteroanterior views (loss of the right II mediastinal arch), posterior displacement of the cardiac silhouette in lateral views, elongation of the left ventricular contour (Snoopy sign), prominent pulmonary artery, radiolucency (lung tissue) between the base of the heart and the diaphragm or between the aortic knuckle and the main pulmonary artery ("tongue" of tissue), or an obscured right heart border by an overlying spine [9].

The diagnosis remains elusive although additional support from radiographic signs, as these features are not specific and may apply to other pathologies.

A careful revaluation of chest radiographic images in the present case shows at least 2 of the described signs (Fig. 1 B): loss of definition of the right cardiac border and a small aortic notch with a prominent incisure. Using CT imaging techniques is important for identifying the diagnosis while excluding other diagnoses in symptomatic patients [4,5].

The abnormal positioning of the heart does not invariably indicate a congenital pericardial defect; hence, other pathological conditions should be considered in the differential diagnosis. Mediastinal displacement can occur in other conditions such as atelectasis, severe pectus excavatum, or pulmonary hypoplasia; the latter often associated with focal hilar lucency due to hyperinflation of the contralateral lung [10].

## Conclusions

In conclusion, the presented case underlines the importance of an integrated imaging approach, with particular on the use of computed tomography to obtain detailed morphological information. The diagnosis of complete pericardial agenesis, although extremely rare, was made possible by the resolution and versatility of angio-CT. The report also illustrates the need to consider rare congenital pathologies in the evaluation of patients with nonspecific cardiac symptoms. This case represents a testimony to the diagnostic challenge, especially when conventional imaging methods do not provide a definitive answer. CT has proven crucial in providing an accurate diagnosis, suggesting that a multimodal approach, including advanced imaging methods, can be crucial in managing complex and unclear cases.

## Patient consent

The patient included in this research gave a written and informed consent to publish the data contained within this study.
